# Physical demands of hernia repair segments and the effect of exoskeleton support

**DOI:** 10.1007/s00464-026-13097-6

**Published:** 2026-07-21

**Authors:** Alec Gonzales, Shuvangee Dhar, Matthew Ball, Alfredo M. Carbonell, Jackie S. Cha

**Affiliations:** 1 Department of Industrial and Systems Engineering, University of Wisconsin-Madison, 3011 Mechanical Engineering, 1513 University Ave, Madison, WI 53706 USA; 2 University of South Carolina School of Medicine Greenville, Greenville, SC USA; 3 Department of Surgery, Prisma Health-Upstate, Greenville, SC USA

**Keywords:** Minimally invasive surgery, Wearables, Work-related musculoskeletal disorders

## Abstract

**Background:**

Previous work has examined the ergonomics of surgical procedures and the effect exoskeletons may have to support surgeons. However, hernia repair procedures are often dynamic, and current literature has yet to deduce physical demands across key segments of these procedures and examine how exoskeletons support surgeons across each of these segments.

**Methods:**

Four surgeons completed a total of 45 open and laparoscopic hernia repair procedures with and without a shoulder-support exoskeleton. Muscle activity and posture during control conditions (i.e., no exoskeleton) were used to characterize the physical demands of hernia repair procedures and were compared to the shoulder-support condition using a linear mixed effects model and t-test respectively.

**Results:**

Mesh deployment was identified as the most demanding segment for open hernia repair procedures and Preparing mesh was the most demanding for laparoscopic procedures. It was found that the shoulder-support exoskeleton reduced muscle activity in the upper trapezius at the expense of increased erector spinae muscle activity and increased neck flexion. During open procedures the shoulder-support exoskeleton largely increased muscle activity across each segment during laparoscopic procedures, but reduced trunk, shoulder, and neck flexion.

**Conclusion:**

The most demanding segments of hernia repair procedures were identified which may be targeted in future interventions to support the physical demands experienced by surgeons. Shoulder-support exoskeletons may be more appropriate for open hernia repair procedures and potentially detrimental for laparoscopic hernia repair procedures.

Hernia repairs are one of the most commonly performed general surgeries in the United States, with over 300,000 performed annually [12]. The physical demands that come with performing the necessary steps of these procedures such as proper mesh placement and fixation may contribute to the potential development of work-related musculoskeletal disorders (WMSDs) in the neck, shoulders, and lower back [9, 21, 23]. However, as these procedures can occur over an extended period of time, there may be more demanding segments of the case which can be greater contributors to potential WMSDs.

Postures adopted during surgery are often considered one of the influencing factors of surgeon physical demands. Since hernia repairs can be performed either open or laparoscopically, physical demands such as muscle activity and neck and flexion angles vary were open cases tend to have higher muscle activity and illicit greater neck and shoulder flexion while laparoscopic cases led to greater neck extension and static postures [18, 19]. However, these studies often evaluate physical demands across the entire case or during select steps rather than distill physical demands to all the specific steps of the case [16, 21].

Passive exoskeletons may support the physical demands and postures of general surgery as previous studies have shown that during surgical procedures they can reduce the time spent in demanding shoulder postures, have led to reduced neck, shoulder, and back discomfort, and have received favorable usability scores from surgeons [3, 8, 10, 15]. However, a current limitation of this work is that results are aggregated across the entire surgical case and do not highlight when during the case the exoskeleton may be most effective or necessary [3].

As such, it is important to identify how these devices influence muscle activity and fatigue during specific segments of the procedure that are characterized by extreme posture. The aims of this project are to (1) identify muscle activity and postures across specific segments of open and laparoscopic hernia repair surgeries and (2) evaluate the effect of a shoulder-support exoskeleton across these segments.

## Methods

### Study population

Forty-five general surgery procedures with two minimally invasive surgical (MIS) surgeons and two MIS fellows from a regional healthcare system were included in this study. All the recruited participants were fellowship trained, minimally invasive surgeons with expertise in hernia repair and all participants completed the informed consent process (IRB 1893300). These participants were recruited through word of mouth and from within the same surgical department. Of the four participants, 75% were male, the average age was 42.3 years (standard deviation [SD] = 8.7), years of experience in general surgery was 10.5 years (SD = 7.2), and 75% were right-handed.

### Surgical procedure

Hernia repair cases were selected as the evaluated surgical procedures due to the high case volume of these procedures at the collaborating regional healthcare institution and because they can be performed using open and laparoscopic techniques. A team of surgeons and human factors engineers identified key segments that occur in open and laparoscopic procedures (Table 1). It should be noted that these steps are not always linear, may repeat, or may not be performed by the evaluated participant (e.g., if the surgeon is instructing the fellow).

**Table 1:** Hernia repair segments

Technique		Segment	Description
Open	Laparoscopic		
–	X	Trocar insertion	Incision and insertion of the trocars
X	X	Access	The creation of the space for hernia dissection
X	X	Hernia dissection	Dissection of the hernia
X	X	Preparing the mesh	Manual measuring and cutting of the mesh
X	X	Mesh deployment	Mesh insertion, stapling, and closing the flap
X	X	Suturing	The use of sutures and closing up of the patient
X	X	Assisting	Static postures holding the laparoscope or retractors

### Equipment and sensors

Electromyography (EMG) sensors (Noraxon USA, Scottsdale, AZ, USA) were used to capture participant muscle activity at 2000 Hz throughout each surgical case. The EMG sensors were prepared and processed according to a standardized procedure [17]. Electrodes were placed on bilateral upper trapezius (UT) [13], medial deltoids (MD) [5], and thoracic and lumbar erector spinae (ES) [7] based on previous work that evaluated these muscles for surgical tasks [3, 4, 20]. Maximum voluntary contractions (MVCs) were gathered for each of the evaluated muscles [1, 5, 13].

The participant’s posture was evaluated with an 11-sensor inertial measurement unit (IMU) system (Movella Holdings Inc., Henderson, NV, USA) that captured their upper body movements. Data was recorded at 60HZ and processed according to the manufacturer’s manual and filtered following a standardized procedure [17, 22]. Trunk and neck flexion and bilateral shoulder flexion and abduction were captured throughout the surgical case.

**Fig. 1:**
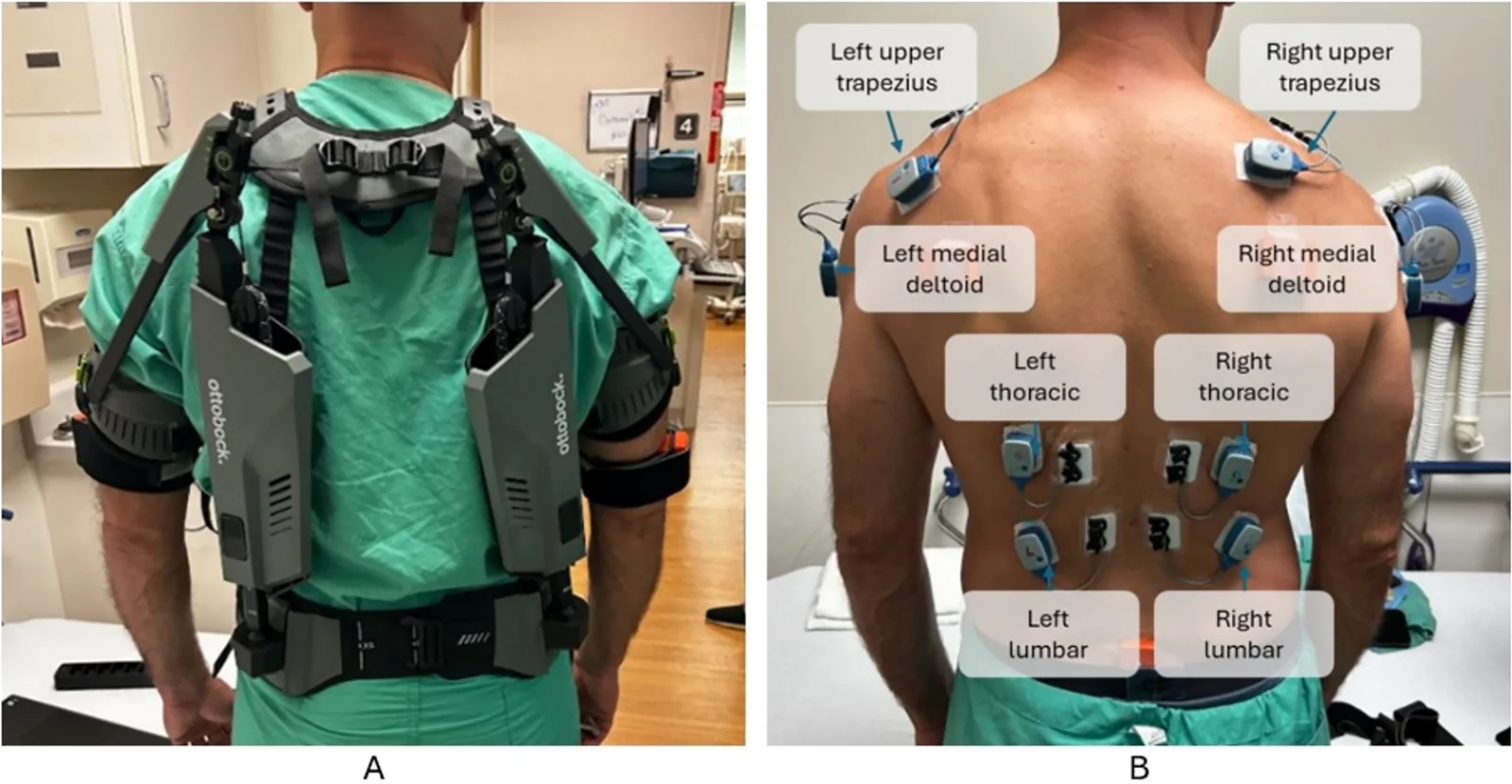
**A** Shoulder-support exoskeleton: Ottobock Shoulder version 03 and **B** the evaluated muscles and electromyography sensor locations

The exoskeleton used in this study (Fig. 1) was the Ottobock Shoulder, version 03 (Ottobock SE & Co. KGaA, Duderstadt, Germany). This exoskeleton was selected as it is designed to support static postures that are adopted during MIS hernia repair procedures [6]. The device was set to its medium setting based on feedback from the participating surgeons.

### Outcome metrics

MVCs were normalized to maximum voluntary exertions (MVEs) and were evaluated based on 10th, median (50th), and 90th percentile (%ile) MVE. Left lumbar and thoracic and right lumbar and thoracic were aggregated to left and right erector spinae (ES) respectively. Time in extreme angles were defined as > 10° neck flexion, > 20° trunk flexion, and > 45° shoulder elevation was considered extreme angles [3, 14].

Self-reported discomfort was gathered after each case using the Borg Rating of Perceived Discomfort (RPD) [11]. Usability of the exoskeleton was reported after each case with the exoskeleton using the System Usability Scale (SUS) [2].

### Study protocol

Before the first case of the study, participants completed the informed consent process and demographic surveys. At the beginning of each study day, electrode locations were identified, prepared, and EMGs were connected to the electrodes. Participants then performed two MVCs for each of the evaluated muscles. MVCs were only gathered before the first case each day. The IMU sensors were then placed on the participant following the standardized upper body–no hands configuration of the system and was calibrated before each case as necessary [22].

Participants were assigned an exoskeleton condition (i.e., shoulder-support or control) at the beginning of the day in a randomized order and completed all their cases for the day (i.e., between two–four cases) in the same exoskeleton condition. During each case, participants were instructed to verbalize which segment of the case they were performing (e.g., suturing). EMG and posture data were gathered throughout the entire case and self-reported discomfort and usability was gathered at the end of each case.

### Data analysis

Descriptive statistics for muscle activity, posture angles, and percentage of time in posture angles were calculated based on Table 1 segments. A linear mixed effects model was used to compare differences in %MVE between exoskeleton conditions while considering: the effects of *case number* (first, second, third, etc.), and *segment occurrence* where the time when the segment occurred during the case was considered (e.g., 10% into the case). Two sample t-tests were completed to compare mean posture angle and mean percent time in extreme posture angles for each segment.

## Results

All participants were included in the data analyses of muscle activity and posture angles. A total of 45 hernia repair cases were included in this study where 25 cases were performed open and 20 were performed laparoscopically. Two cases converted from laparoscopic to open and one case included both an inguinal and ventral procedure. Each of these cases were analyzed according to their respective open and laparoscopic conditions. Open and laparoscopic inguinal cases were performed using the Standard Open Anterior Mesh Repair and Laparoscopic Transabdominal Preperitoneal techniques respectively; Open and laparoscopic ventral cases were performed using the Open Retromuscular Ventral Hernia Repair and Laparoscopic Intraperitoneal Onlay Mesh techniques respectively. Descriptives of time in seconds for each segment of open and laparoscopic surgical cases and descriptives of case types can be found in Tables 2 and 3 respectively.

**Table 2:** Hernia repair surgical case descriptives

Procedure	Segment	Time–minutes, Mean (SD)	Time–minutes Min / Max	Percentage of case, Mean (SD)
Open *n* = 25	Access	31.9 (29.6)	3.1 / 93.7	35 (22)
	Hernia dissection	15.5 (17.1)	1.6 / 61.6	18.6 (15.2)
	Preparing mesh	2.2 (1.1)	1 / 4.4	2.6 (2.5)
	Mesh deployment	6.2 (4.5)	0.6 / 14	8 (8.5)
	Suturing	24.4 (27.5)	1.5 / 102.4	31.2 (35)
	Assisting	30 (30.2)	0.7 / 99.9	37.7 (25.5)
	Total case length	107.5 (61.8)	15.03 / 245.3	
Laparoscopic *n* = 20	Trocar insertion	6.6 (2.9)	2.5 / 12.1	13.6 (7.7)
	Access	8.3 (4.7)	1.7 / 16.2	17.8 (13.6)
	Hernia dissection	17.5 (12.1)	0.8 / 47.6	29.6 (20.5)
	Preparing the mesh	1.9 (1)	0.8 / 4.5	3.8 (1.8)
	Mesh deployment	8.5 (7.1)	1 / 20.6	15.8 (12.7)
	Suturing	8.57 (12.83)	1 / 39.9	15.3 (19)
	Assisting	30.71 (24.27)	1.6 / 58.1	43.7 (29.6)
	Total case length	59.6 (25.6)	15.1 / 137.2

**Table 3:** Hernia repair procedures

Procedure	Total operations*, n*	Open cases, *n*		Laparoscopic cases, *n*	
		Control	Exoskeleton	Control	Exoskeleton
Inguinal	20	0	1	12	7
Ventral	25	11	13	0	1

### Muscle activity across cases

#### Open hernia repair cases

**Fig. 2:**
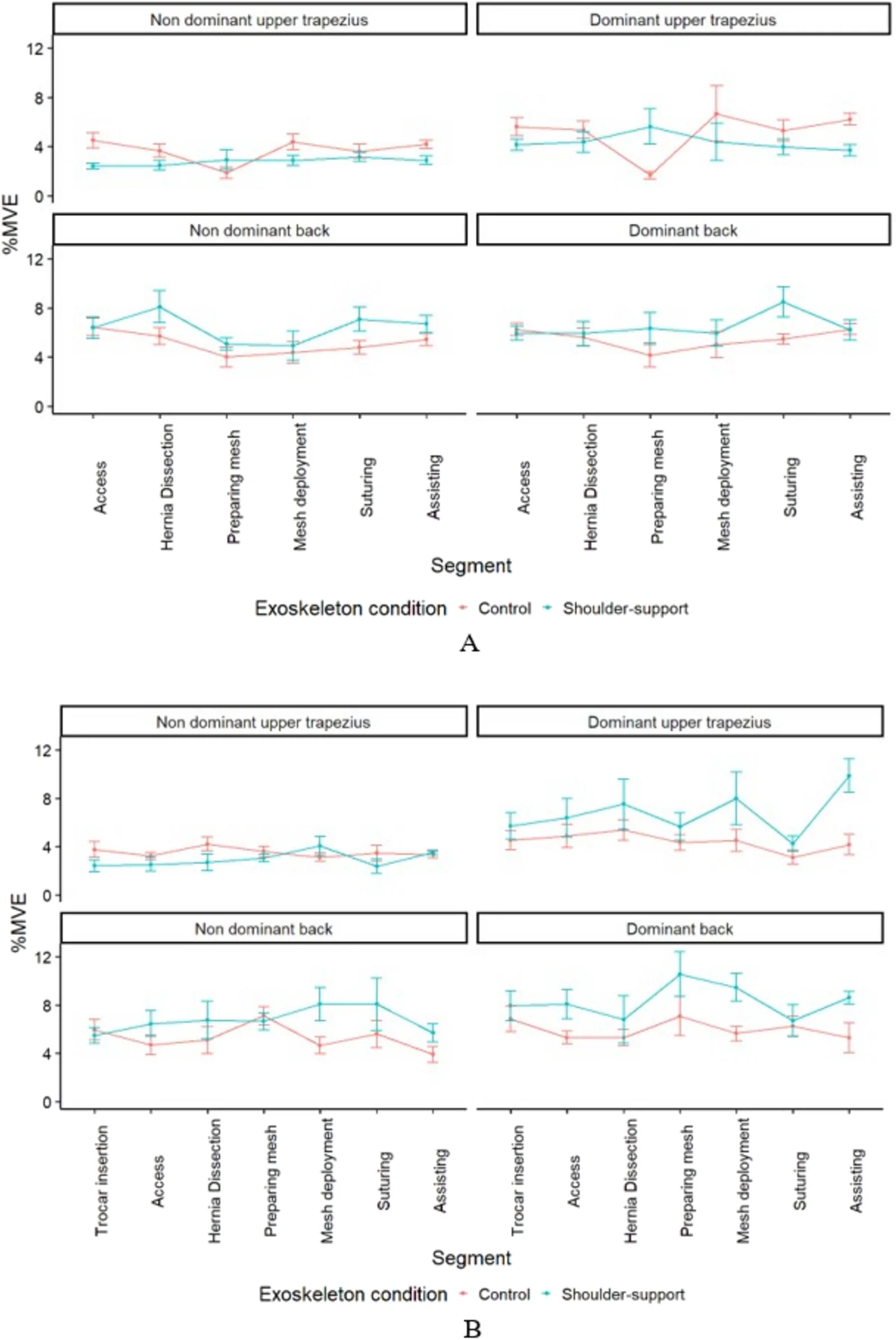
Median muscle activity of bilateral upper trapezius and back for all participants **A** open hernia repair and **B** laparoscopic hernia repair case. Error bars represent standard error

Median %MVE results for open cases can be found in (Fig. 2A) and descriptives can be found in Table 4. Median muscle activity was largest for the dominant UT and non-dominant ES during Hernia dissection, non-dominant and dominant MD during Access, non-dominant UT during Assisting, and dominant ES during Mesh deployment.

**Table 4:** Descriptives (Mean, Standard Deviation (SD), Range, and Percents) of muscle activity across open and laparoscopic hernia repair segments

Open hernia repair						
Muscle	Segment	Statistic	Control %MVE (SD)	Exoskeleton %MVE (SD)	Control %MVE Range	Exoskeleton %MVE Range
Dominant upper trapezius	Access	10th %ile	1.4 (1.8)	1.2 (1.8)	0.2–6.1	0.3–5.5
		50th %ile	4.8 (3.5)	4.4 (2.3)	0.8–12	0.6–7.8
		90th %ile	11 (5.4)	8.5 (3.6)	2.7–21	1.9–17.2
	Hernia dissection	10th %ile	2.8 (2)	0.8 (2.4)	0.2–5.7	0.2–6.2
		50th %ile	5.7 (2.9)	4 (3.1)	0.7–9.4	0.6–9.2
		90th %ile	10.7 (4.3)	9 (4.6)	3.4–21.5	2.1–16
	Preparing mesh	10th %ile	0.4 (0.3)	3.5 (1.8)	0.1–0.9	0.1–4.4
		50th %ile	1.8 (0.8)	6.5 (3.2)	0.3–2.5	0.4–9.1
		90th %ile	4.9 (5.6)	9.4 (3.3)	0.9–17	4–13.2
	Mesh deployment	10th %ile	0.7 (3.5)	0.7 (2.6)	0.3–9.1	0.2–5.9
		50th %ile	5.1 (5.5)	2.1 (4)	1.8–16.8	0.5–10.5
		90th %ile	12.8 (7.8)	6.6 (5)	5.6–27.2	1.8–15.8
	Suturing	10th %ile	1.2 (2.8)	1.1 (2.1)	0.2–8.9	0.3–7.7
		50th %ile	4.3 (4.1)	3.7 (2.9)	0.9–14.2	0.8–11.4
		90th %ile	10.1 (5.5)	8 (4.1)	1.9–22.2	2.7–17.3
	Assisting	10th %ile	2.4 (2.2)	0.8 (1.9)	0.2–7.8	0.2–7.2
		50th %ile	6.1 (3.1)	2.7 (2.8)	0.8–13.2	0.6–10
		90th %ile	11.8 (4.6)	7.4 (4.5)	3.7–21	1.5–21.7
Non-dominant upper trapezius	Access	10th %ile	1.4 (0.9)	1 (0.5)	0.1–3.1	0.2—1.9
		50th %ile	4.2 (3)	2.3 (1.1)	0.6–11.2	0.5–4.4
		90th %ile	7.8 (5.9)	5 (2.5)	2.6–22.6	2–12.1
	Hernia dissection	10th %ile	0.9 (1.3)	1 (0.8)	0.2–4	0.2–2.8
		50th %ile	3.1 (2.2)	2.7 (1.4)	0.8–7.3	0.3–4.8
		90th %ile	7.7 (3.7)	5.2 (3.4)	2.7–17.9	1.7–13.2
	Preparing mesh	10th %ile	0.3 (0.6)	1.2 (0.8)	0.1–1.5	0.1–1.9
		50th %ile	1.8 (1.1)	3.3 (1.8)	0.3–3.3	0.5–5.4
		90th %ile	4.7 (2.5)	5.5 (1.2)	1–8.5	5.2–8.2
	Mesh deployment	10th %ile	1.2 (1.1)	0.7 (0.7)	0.4–2.9	0.2–2.2
		50th %ile	3.9 (1.6)	2.7 (1.1)	2.8–6.7	1–4.6
		90th %ile	10.2 (2.5)	5.7 (2.9)	7.1–13.3	2.7–10.2
	Suturing	10th %ile	0.9 (1.5)	1 (1)	0.1–5.5	0.2–4
		50th %ile	2.7 (2.7)	2.9 (1.7)	0.7–10.4	0.6–6.8
		90th %ile	5.3 (4.8)	6.2 (4.3)	2.8–18.2	2.5–19.1
	Assisting	10th %ile	1.2 (1.4)	0.8 (0.9)	0.1–4.6	0.2–3.8
		50th %ile	4.3 (2.2)	2.4 (2)	0.3–8.4	0.6–10.2
		90th %ile	8.4 (4.2)	6.5 (3.4)	2.3–18.1	1.8–16.3
Dominant medial deltoid	Access	10th %ile	0.2 (0.1)	0.21 (0.2)	0.1–0.3	0.1–0.7
		50th %ile	0.9 (0.3)	0.9 (0.9)	0.2–1.4	0.2–2.9
		90th %ile	4.4 (2)	4.2 (3.1)	0.7–10.4	1.1–11.8
	Hernia dissection	10th %ile	0.2 (0.1)	0.3 (0.2)	0.1–0.3	0.1–0.6
		50th %ile	0.8 (1.6)	1.2 (1.1)	0.2–7.2	0.2–3.3
		90th %ile	3.5 (3.4)	5.8 (4.3)	0.5–14.1	0.8–12.7
	Preparing mesh	10th %ile	0.1 (0)	0.2 (0.1)	0.1–0.2	0.1–0.3
		50th %ile	0.5 (0.3)	0.7 (0.5)	0.1–1.1	0.2–1.5
		90th %ile	3.2 (2.3)	5.4 (2.2)	0.3–6.8	1.5–7
	Mesh deployment	10th %ile	0.1 (0.1)	0.2 (0.1)	0.1–0.4	0.1–0.3
		50th %ile	0.5 (1.1)	0.6 (0.8)	0.2–3	0.2–2.3
		90th %ile	4.5 (4.5)	2.1 (3.2)	1.8–13.9	0.5–9
	Suturing	10th %ile	0.2 (0.1)	0.2 (0.1)	0.1–0.4	0.1–0.6
		50th %ile	0.8 (0.6)	0.8 (1.1)	0.2–2.5	0.2–3.9
		90th %ile	3.3 (2.6)	4.2 (3.1)	1–9	1.2–13.3
	Assisting	10th %ile	0.2 (0.1)	0.2 (0.1)	0.1–0.7	0.1–0.6
		50th %ile	0.8 (0.8)	0.5 (0.9)	0.2–3.9	0.2–3.6
		90th %ile	4.7 (3.4)	3.3 (2.6)	0.8–14.5	0.8–9.7
Non-dominant medial deltoid	Access	10th %ile	0.2 (0.1)	0.2 (0.2)	0–0.5	0.1–0.7
		50th %ile	0.5 (0.6)	0.5 (0.5)	0–2	0.2–1.9
		90th %ile	3 (2.5)	2.7 (2.5)	0–8.7	0.8–10.7
	Hernia dissection	10th %ile	0.2 (0.2)	0.3 (0.1)	0–0.9	0.1–0.6
		50th %ile	0.5 (0.6)	0.5 (0.5)	0–2.2	0.1–1.9
		90th %ile	3.9 (3.3)	2.6 (2.2)	0–13.6	0.57–8.3
	Preparing mesh	10th %ile	0.1 (0.1)	0.2 (0.1)	0.1–0.2	0.1–0.4
		50th %ile	0.3 (0.3)	1 (0.5)	0.1–0.9	0.3–1.5
		90th %ile	2 (3.1)	3.2 (2)	0.3–8.9	1.82–6.4
	Mesh deployment	10th %ile	0.1 (0.1)	0.2 (0.1)	0.1–0.2	0.1–0.4
		50th %ile	0.4 (0.4)	0.4 (0.4)	0.2–1.3	0.2–1.1
		90th %ile	2.7 (3.5)	3.2 (1.5)	1.2–9.8	0.7–4.8
	Suturing	10th %ile	0.1 (0.2)	0.2 (0.1)	0–0.6	0.1–0.5
		50th %ile	0.3 (0.7)	0.8 (1)	0–2.2	0.3–3.7
		90th %ile	1.8 (2.6)	4.2 (3.8)	0–10	1.5–15.2
	Assisting	10th %ile	0.2 (0.1)	0.2 (0.1)	0–0.6	0.1–0.5
		50th %ile	0.4 (0.4)	0.5 (0.5)	0–1.9	0.1–3
		90th %ile	2.6 (2.6)	2.7 (3.5)	0–12.1	0.6–18.4
Dominant back	Access	10th %ile	2.5 (1.7)	2.5 (1.6)	0.9–7.5	1.3–6.5
		50th %ile	5.7 (2.4)	6.1 (2.9)	2.5–11.6	1.9–11.5
		90th %ile	9.9 (3.1)	9.1 (4.5)	4.4–17.5	3.2–19.5
	Hernia dissection	10th %ile	2.8 (1.9)	2.3 (2.2)	0.9–8.7	1.2–8.6
		50th %ile	5.8 (3)	4.7 (3.8)	1.4–14.1	1.8–12.8
		90th %ile	8.8 (3.6)	9.3 (6.8)	3.7–18.1	2.8—24.5
	Preparing mesh	10th %ile	1.9 (1.4)	2.3 (1.4)	0.8–4.2	1.5–5
		50th %ile	3.9 (2.4)	6.8 (2.9)	1.8–7.3	2.5–9.7
		90th %ile	6.6 (3.2)	10 (4.1)	3.3–12	3.3–13
	Mesh deployment	10th %ile	2.9 (1.4)	2.5 (1.9)	0.8–4.1	1.3–5.5
		50th %ile	6 (2.6)	6.8 (2.8)	1.7–7.4	2.4–9.4
		90th %ile	9.5 (4.5)	10.8 (4.9)	3–15	3.9–18.9
	Suturing	10th %ile	3.1 (1.5)	2.9 (3.4)	0.7–6.4	1.3–13.9
		50th %ile	5.3 (1.9)	7.8 (5.6)	0.9–8.6	1.7–21.3
		90th %ile	8.5 (2.4)	11.5 (8.5)	2.5–13.1	2.8–30.5
	Assisting	10th %ile	3 (2.1)	1.9 (3)	0.8–12.2	1.1–11.8
		50th %ile	5.6 (2.9)	3.8 (4.9)	1.3–15.6	1.5–17.7
		90th %ile	9.8 (3.3)	8.3 (6.9)	2.8–19	1.9–27.1
Non-dominant back	Access	10th %ile	2.6 (1.7)	2.53 (2.7)	0.6–7.1	0.7–13.4
		50th %ile	6.2 (3.5)	6.1 (4.2)	0.9–14.1	1.2–19.3
		90th %ile	9.2 (5.7)	8.6 (5.7)	2–20.7	3.4–26.8
	Hernia dissection	10th %ile	2.8 (2)	3.1 (2.5)	0.5–9.1	1.4–9.8
		50th %ile	6.2 (2.8)	7.3 (4.9)	0.7–11.6	2.36–17.5
		90th %ile	9.1 (4.2)	10.7 (7.4)	1.5–15.2	3.9–26.3
	Preparing mesh	10th %ile	1.6 (0.9)	2.9 (1.1)	0.5–3	1–3.5
		50th %ile	4.6 (1.96)	5.12 (1.14)	0.68–5.79	3.9–6.57
		90th %ile	8.5 (3.5)	8.2 (1.1)	1.3–10.6	5.9–8.5
	Mesh deployment	10th %ile	1.7 (1.2)	2.1 (1.1)	0.6–3.9	0.7–3.5
		50th %ile	4.2 (2.1)	3.8 (3.1)	1–7.4	1.9–11
		90th %ile	8.9 (3.1)	10.3 (4.9)	2.5–11.4	3.9–18.3
	Suturing	10th %ile	2.3 (1.4)	2.5 (2)	0.5–5.2	0.7–7.1
		50th %ile	4.8 (2.6)	5.2 (4.5)	0.7–8.7	1.6–16.7
		90th %ile	7.7 (4.1)	8.7 (6.9)	1.2–16.7	4.1–25.4
	Assisting	10th %ile	2.2 (1.4)	2.4 (2.3)	0.5–6.7	0.8–9.03
		50th %ile	5.3 (3.3)	5.7 (4.3)	0.7–15.8	1.5–15.8
		90th %ile	8.4 (5)	8.7 (6.3)	1.5–23.3	2.8–25.9

Laparoscopic hernia repair						
Muscle	Segment	Statistic	Control %MVE (SD)	Exoskeleton %MVE (SD)	Control %MVE Range	Exoskeleton %MVE Range
Dominant upper trapezius	Trocar insertion	10%ile	0.8 (1.9)	2.7 (2)	0.3–5.8	0.2–5.8
		50%ile	4.4 (2.5)	5.6 (2.7)	1.7–9.7	1.9–9.9
		90%ile	9.4 (4.3)	11.4 (3.6)	5.5–19	5.4–15.8
	Access	10%ile	1.9 (2.8)	4.2 (3)	0.1–8.5	0.1–9.4
		50%ile	3.6 (3.7)	6.3 (4.5)	0.3–12.1	0.4–14.4
		90%ile	7.5 (4.9)	9.6 (5.4)	1.2–16.8	1.9–19.8
	Hernia dissection	10%ile	1.8 (2.3)	4 (3.9)	0.3–7.8	1–11.1
		50%ile	4.3 (2.9)	6.3 (4.6)	2.2–11.2	2.9–15.3
		90%ile	9.2 (3.7)	9.2 (5.7)	4–16	6.5–21
	Preparing mesh	10%ile	0.9 (1.1)	1.1 (1.6)	0.1–3.6	0.2–5
		50%ile	4.5 (2.5)	6.2 (3)	0.8–7.4	0.7–8.9
		90%ile	9.7 (3.7)	11.1 (4.4)	2–15.1	1.8–14.2
	Mesh deployment	10%ile	0.9 (1.7)	2.7 (3.9)	0.1–6.2	0.1–11.8
		50%ile	3.6 (3.4)	7.5 (6.2)	0.4–10.5	0.4–18.2
		90%ile	6.8 (4.1)	11.2 (8.7)	2.6–15.5	3.3–28.6
	Suturing	10%ile	0.7 (1.2)	2.4 (1.4)	0.2–4.1	0.4–3.4
		50%ile	3 (1.8)	4.8 (1.4)	0.6–7.4	1.8–5.3
		90%ile	6.4 (3.3)	8.4 (2.2)	3–12.9	6.7–12.7
	Assisting	10%ile	1.3 (2)	7.2 (3.7)	0.2–5.8	0.3–10.4
		50%ile	3.1 (2.7)	11.3 (4.4)	1.4–9.9	1.7–14.8
		90%ile	6.4 (4)	16.9 (5.5)	4.8–16.6	3.3–20.7
Non-dominant upper trapezius	Trocar insertion	10%ile	1.6 (0.7)	0.9 (0.6)	0.4–2.4	0.3–1.9
		50%ile	3.3 (2.2)	2.3 (1.2)	1.9–9.8	1.1–4
		90%ile	6.2 (5)	5.7 (2.7)	4.4–20.5	2.6–8.7
	Access	10%ile	2.1 (0.9)	1.1 (1)	0.4–3.1	0.2–2.9
		50%ile	3.1 (1.1)	1.8 (1.6)	1.5–5	0.9–5.3
		90%ile	5.8 (2.6)	3.7 (2.2)	2.5–11.7	2–7.8
	Hernia dissection	10%ile	2.1 (1.3)	1.2 (1.1)	0.3–4.9	0.4–2.8
		50%ile	3.8 (2)	1.9 (1.5)	1.3–8.1	1.3–4.9
		90%ile	7.5 (3.1)	3.6 (2)	3.4–13.7	2.6–7.6
	Preparing mesh	10%ile	1.2 (0.9)	1.2 (0.5)	0.1–2.9	0.2–1.8
		50%ile	3.9 (1.7)	3.1 (0.8)	1.5–7.5	1.9–4.4
		90%ile	6.6 (2.2)	5 (1.4)	4.4–12.7	4.3–8.2
	Mesh deployment	10%ile	1 (0.6)	1.9 (1.6)	0.2–2.2	0.3–4.2
		50%ile	3.1 (1.3)	4.4 (2.2)	1.4–6.6	1.2–6.6
		90%ile	6.2 (2.7)	8.4 (2.7)	3–13.5	2.6–10.5
	Suturing	10%ile	1 (1)	0.8 (0.6)	0.2—2.7	0.4–1.6
		50%ile	2.9 (2.1)	2.4 (1.3)	1–7.4	1.1–4.4
		90%ile	5.8 (4.6)	3.4 (4.2)	3.4–18.2	2.6–12.6
	Assisting	10%ile	1.7 (0.6)	1.8 (0.9)	0.6–2.3	0.2–2.8
		50%ile	3.4 (0.9)	3.7 (0.6)	2–5.2	2.4–4.3
		90%ile	5.6 (2.8)	5.6 (0.8)	3.5–12.6	4.6–6.8
Dominant medial deltoid	Trocar insertion	10%ile	0.2 (0.1)	0.2 (0.1)	0.1–0.3	0.1–0.2
		50%ile	0.6 (0.8)	0.6 (0.6)	0.2–2.7	0.4–1.9
		90%ile	8.5 (3.5)	4.5 (1.4)	2.3–11.6	2.5–6.3
	Access	10%ile	0.2 (0.1)	0.3 (0.5)	0.1–0.6	0.1–1.7
		50%ile	0.4 (1.2)	1.8 (1.4)	0.1–4.9	0.1–4.5
		90%ile	2 (3.3)	5.8 (3)	0.2–10.8	0.3–8.1
	Hernia dissection	10%ile	0.2 (0.1)	0.4 (1.4)	0.1–0.5	0.1–3.5
		50%ile	0.7 (0.8)	1.4 (2.3)	0.2–3.1	0.3–6
		90%ile	4.2 (2.2)	4.6 (2.8)	0.2–7.4	1.5–9.2
	Preparing mesh	10%ile	0.1 (0.3)	0.2 (0)	0.1–1.4	0.2–0.2
		50%ile	0.6 (1.1)	0.8 (0.3)	0.1–4.4	0.7–1.4
		90%ile	5.1 (2.3)	4.7 (1.1)	0.5–8.6	2.5–5.5
	Mesh deployment	10%ile	0.2 (0)	0.2 (0.9)	0.1–0.2	0.1–2.7
		50%ile	0.5 (0.4)	0.7 (3.7)	0.1–1.8	0.2–10.5
		90%ile	2.8 (2)	3.4 (7.1)	0.7–8	0.5–21.7
	Suturing	10%ile	0.2 (0)	0.3 (0.1)	0.1–0.3	0.2–0.5
		50%ile	0.6 (0.3)	1.3 (0.8)	0.2–0.9	0.2–2.2
		90%ile	3.3 (6.6)	4.5 (2.4)	0.8–22.9	3.3–9.2
	Assisting	10%ile	0.1 (0.1)	0.4 (0.2)	0.1–0.4	0.1–0.8
		50%ile	0.4 (0.5)	1.9 (1.2)	0.2–1.9	0.2–3.6
		90%ile	2.5 (2.9)	5.4 (2.3)	0.8–9	1.1–6.8
Non-dominant medial deltoid	Trocar insertion	10%ile	0.2 (0.1)	0.2 (0)	0.1–0.5	0.1–0.2
		50%ile	0.3 (0.6)	0.4 (0.2)	0.2–2.4	0.2–0.7
		90%ile	3 (2.6)	2.3 (1.9)	1.1–9	0.9–6.2
	Access	10%ile	0.2 (0.4)	0.2 (0.1)	0.1–1.6	0.2–0.4
		50%ile	0.7 (1.3)	0.5 (1)	0.1–4	0.3–2.9
		90%ile	4.3 (2.4)	2.1 (2.1)	0.2–7.3	0.4–5.9
	Hernia dissection	10%ile	0.3 (0.3)	0.2 (0.1)	0.1–0.9	0.2–0.4
		50%ile	1 (1)	0.8 (0.9)	0.3–2.9	0.2–2.3
		90%ile	4 (1.7)	2.7 (2.6)	1.1–6.4	0.3–6.4
	Preparing mesh	10%ile	0.2 (0.1)	0.2 (0.1)	0.1–0.5	0.1–0.4
		50%ile	1.3 (1.1)	0.6 (0.2)	0.2–3.7	0.3–0.7
		90%ile	5.6 (2.6)	2.7 (0.9)	1.2–10.9	1.4–4
	Mesh deployment	10%ile	0.2 (0.1)	0.3 (0.1)	0.1–0.6	0.1–0.4
		50%ile	0.4 (0.8)	0.6 (0.7)	0.2–2.5	0.4–2.2
		90%ile	3.2 (1.5)	2.7 (2.1)	1.7–6.7	0.8–7.2
	Suturing	10%ile	0.2 (0.2)	0.3 (0.1)	0.1–0.7	0.2–0.4
		50%ile	0.4 (1.1)	0.7 (0.8)	0.2–3	0.5–2.6
		90%ile	2.7 (2.9)	4.2 (3.5)	0.5–9.9	2.2–11
	Assisting	10%ile	0.2 (0.1)	0.2 (0)	0.1–0.3	0.1–0.2
		50%ile	0.3 (3.4)	0.3 (0)	0.2–11.1	0.2–0.4
		90%ile	2 (5)	0.6 (0.2)	1.2–17.2	0.5–1.1
Dominant back	Trocar insertion	10%ile	4.1 (2.1)	5.1 (2)	0.8–6.2	1.8–6.3
		50%ile	7.2 (3.5)	8.8 (3)	2.1–13.4	2.5–10.5
		90%ile	10.4 (5.2)	14.8 (4.7)	3.8–20.4	3.5–15.7
	Access	10%ile	3.4 (1.7)	5.2 (2.8)	0.6–6.3	1.7–9.4
		50%ile	5.5 (2)	8.1 (3.4)	0.8–8.2	2.1–12.2
		90%ile	9.2 (2.9)	11 (4.3)	1.8–11.9	2.6–15.9
	Hernia dissection	10%ile	3.3 (2)	6 (3.3)	1.2–7	1.6–9.1
		50%ile	5.1 (2.4)	8.5 (4.4)	1.9–9.2	1.9–12.2
		90%ile	8.8 (2.9)	11.4 (5.4)	3.6–12	2.3–15.9
	Preparing mesh	10%ile	2.8 (2)	5.8 (2.6)	0.6–8.3	1.6–8.8
		50%ile	6.1 (6.3)	11.3 (4.9)	0.9–26.7	2.2–16.5
		90%ile	9.3 (11.9)	15.9 (6.3)	1.4–52	2.6–21.3
	Mesh deployment	10%ile	3.7 (1.8)	5.1 (2.5)	0.9–6.3	1.9–9.4
		50%ile	5.8 (2.3)	10.6 (3.3)	2.3–9.6	4.1–13.3
		90%ile	10.1 (3.4)	14.4 (3.4)	4.7–17.4	8.6–18.4
	Suturing	10%ile	3.3 (2.2)	3.8 (2)	1–8.4	1.5–6.3
		50%ile	6.1 (2.7)	7.5 (3)	2.1–10.4	2–9.8
		90%ile	9.4 (3.7)	10.5 (4.8)	3.8–15.4	2.6–15.9
	Assisting	10%ile	3.4 (2.3)	4.6 (1.7)	0.7–7.1	2–7.9
		50%ile	5.1 (3.9)	7.9 (1.6)	1.2–14	6.9–11.8
		90%ile	8.9 (5)	12.9 (2.3)	3.1–18.5	12.4–19
Non-dominant back	Trocar insertion	10%ile	1.9 (1.3)	3.3 (1.2)	0.9–5.2	1.5–4.7
		50%ile	5.8 (2.8)	5.5 (1.6)	2.4–10.6	3.4–8.1
		90%ile	10.9 (4.3)	8.6 (2.8)	6.1–18.3	5.3–12.5
	Access	10%ile	2.3 (2)	4.4 (1.7)	0.7–7.7	1.4–6.5
		50%ile	4.7 (3.2)	7.5 (3)	0.9–11.2	2.5–11
		90%ile	6.5 (4.3)	10.3 (3.8)	2.2–17.9	3.8–15.5
	Hernia dissection	10%ile	2.1 (2.2)	6.1 (2.2)	0.7–6.6	1.9–6.4
		50%ile	3.9 (3.9)	7.9 (3.5)	0.9–11.5	2.7–11.1
		90%ile	5.9 (5.2)	9.6 (4.8)	2–17.8	3.5–15.9
	Preparing mesh	10%ile	3.1 (1.9)	3.9 (1.3)	0.9–7.4	1.7–5.9
		50%ile	6.6 (2.8)	6.6 (1.9)	2.5–12.7	3.3–9.2
		90%ile	10.2 (3.4)	9.7 (3.2)	6.3–17.3	5.1–15
	Mesh deployment	10%ile	1.9 (1.2)	4 (2.2)	0.7–5.6	2.1–8.1
		50%ile	3.9 (2.5)	6.9 (3.9)	1–9.8	4.2–14.1
		90%ile	6.9 (3.9)	10.9 (4.8)	2–17	7–19.4
	Suturing	10%ile	2.4 (2.2)	3.3 (1.3)	0.6–7.1	1.9–5.4
		50%ile	4.3 (3.6)	5.9 (4.9)	1.1–13.1	3.4–13.8
		90%ile	8.2 (6.9)	9.7 (7.1)	2.1–27	4.8–21.2
	Assisting	10%ile	1.7 (1.1)	4.2 (1.7)	0.8–4.1	1.1–6.5
		50%ile	3.5 (2.1)	6.4 (2.4)	1.2–8.1	2–9
		90%ile	7.4 (3.1)	9.3 (2.1)	3.5–13.7	5.1–11.4

#### Effects of exoskeleton

During Access, compared to control, there was a reduction in the non-dominant UT (*β* = −1.57, *p* < 0.01) and an increase in the dominant MD (*β* = 0.52, *p* < 0.01). However, there was an increase in the non-dominant ES during Hernia dissection (*β* = −0.33, *p* < 0.01) and Suturing (*β* = 1.63, *p* = 0.04). For Assisting, there was a decrease in dominant UT (*β* = −1.20, *p* = 0.02), an increase in non-dominant MD (*β* = 0.20, *p* = 0.05), and an increase in the non-dominant ES %MVE (*β* = 1.38, *p* = 0.02).

#### Effects of case number and segment occurrence

Considering the fixed effects, as case number increased there was a decrease in %MVE during Preparing mesh (*β* = −12.97, *p* = 0.04), Suturing (*β* = −5.40, *p* = 0.04), and the non-dominant back %MVE during Assisting (*β* = −2.72, *p* < 0.01). As segment occurrence increased, %MVE decreased during Suturing (*β* = −0.15, *p* = 0.04), and in the non-dominant ES during Assisting (*β* = −0.11, *p* < 0.01), but %MVE increased in the dominant MD during Assisting (*β* = 0.20, *p* < 0.01). There was a significant interaction between case number and segment occurrence for %MVE during Preparing mesh (*β* = 0.17, *p* = 0.04), and for the dominant MD (*β* = 10.38, *p* = 0.03) and non-dominant back (*β* = 0.03, *p* < 0.01) during Assisting.

#### Laparoscopic hernia repair cases

Median %MVE results for laparoscopic cases can be found in (Fig. 2B) and descriptives can be found in Table 4. Median muscle activity was largest for the non-dominant and dominant UT, non-dominant MD, and non-dominant ES during Preparing mesh, and dominant MD and ES during Hernia dissection.

#### Effects of exoskeleton

For Hernia dissection, compared to control, there was an increase in dominant MD %MVE (*β* = 1.92, *p* = 0.03). Additionally, for Mesh deployment, there was an increase in non-dominant ES %MVE (*β* = 4.66, *p* < 0.01), and an increase in dominant ES %MVE (*β* = 2.76, *p* = 0.02). For Assisting, there was an increase in non-dominant back %MVE (*β* = 2.71, *p* = 0.03).

#### Effects of case number and segment occurrence

Considering the fixed effects, as case number increased %MVE increased in the dominant UT (*β* = −2.27, *p* = 0.02) during Preparing mesh, but %MVE decreased in the non-dominant ES during Mesh deployment (*β* = −4.27, *p* = 0.03), and in the non-dominant MD (*β* = −1.97, *p* = 0.02) and non-dominant ES (*β* = −3.92, *p* = 0.03) during Suturing. As segment occurrence increased, %MVE decreased in the non-dominant back (*β* = −0.13, *p* = 0.04) during Mesh deployment, non-dominant MD (*β* = −0.05, *p* = 0.01) and non-dominant ES (*β* = −0.10, *p* = 0.03) during Suturing, but %MVE increased in the dominant ES (*β* = 0.12, *p* = 0.03) during Mesh deployment. A significant interaction was found between case number and segment occurrence the non-dominant MD %MVE (*β* = 0.02, *p* = 0.03) during Suturing.

### Posture angles across cases

#### Open hernia repair cases

**Fig. 3:**
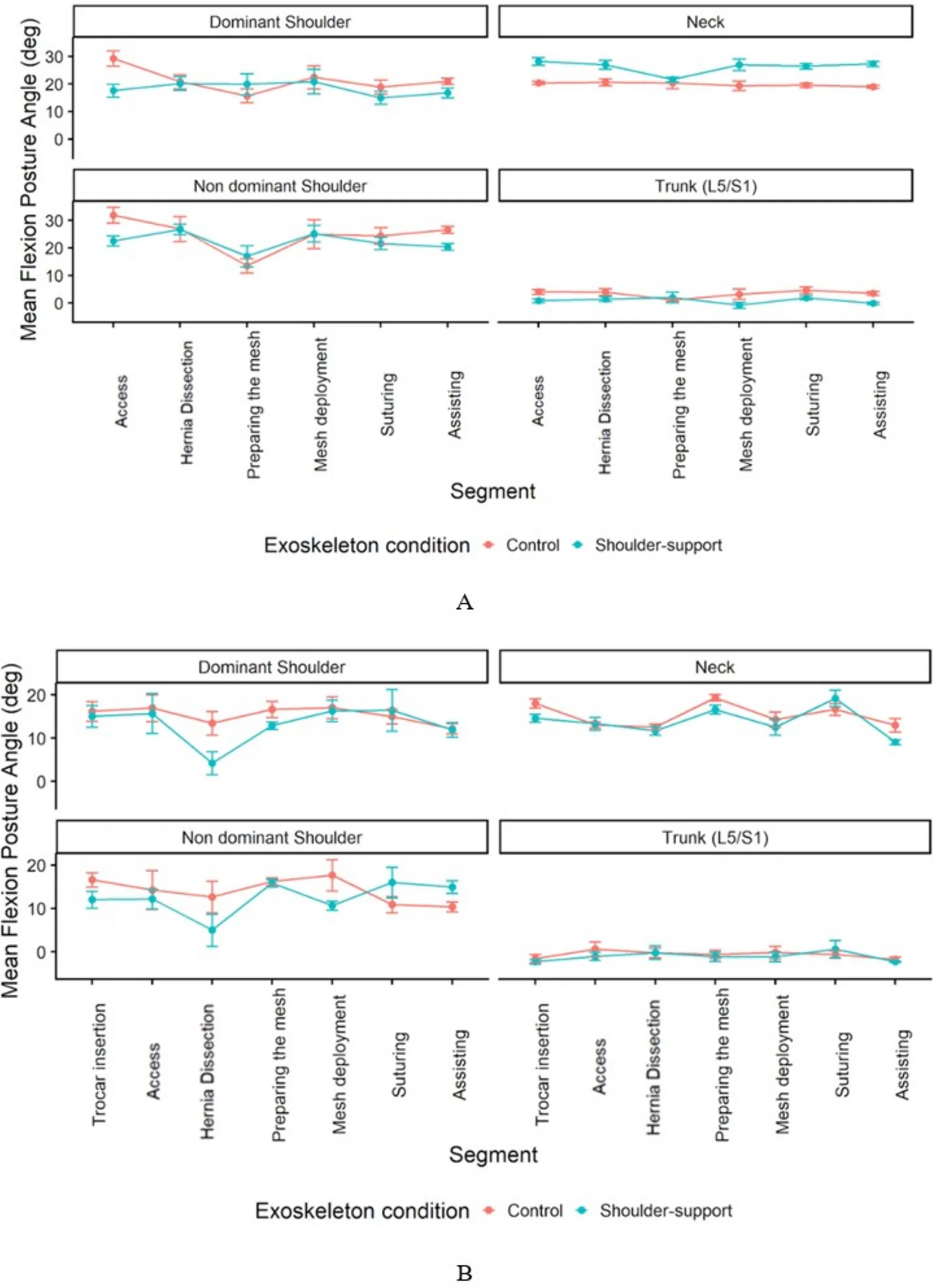
Mean trunk, shoulder, and neck posture angles across **A** open hernia repair and **B** laparoscopic hernia repair segments. Error bars represent standard error

Mean posture results for open cases can be found in (Fig. 3A) and descriptives can be found in Table 5. The largest non-dominant and dominant shoulder flexion was during Assisting and Access. The longest mean percentage of time spent in extreme non-dominant and dominant shoulder postures was during Mesh deployment. The largest neck and trunk flexion were observed during Hernia dissection and Suturing. The longest mean percentage of time spent in extreme neck and trunk angles was during Mesh deployment and Hernia dissection.

**Table 5:** Descriptive Mean, Standard Deviation (SD), and Range of posture and percent of time spent in extreme posture angles across open and laparoscopic hernia repair segmentst

Open hernia repair							
Posture Angle	Segment	Control Mean (SD)	Exoskeleton Mean (SD)	Control Range	Exoskeleton Range	Control Percent	Exoskeleton Percent
Neck flexion	Access	20.4 (3.8)	28.2 (2.9)	0.8–27.8	5.3–34.4	89.8	89.9
	Hernia dissection	20.6 (3.6)	26.9 (2.3)	1.7–27.1	10.7–32.0	82.0	86.5
	Suturing	19.6 (3.1)	26.4 (3.1)	− 2.7–26.3	6.2–35.2	83.4	91.1
	Preparing the mesh	20.4 (3.5)	21.6 (4.4)	9.8–26.2	6.7–26.5	100.0	69.6
	Mesh deployment	19.3 (4.2)	27.0 (3.9)	3.1–28.2	7.8–33.8	100.0	85.7
	Assisting	19.0 (4.3)	27.3 (3.9)	− 4.0–28.3	4.0–36.1	87.1	73.9
	Total	19.6 (4.7)	26.9 (3.1)	− 8.8–29.7	1.0–36.3	100.0	93.0
Trunk flexion (L5/S1)	Access	4.0 (3.0)	0.8 (2.0)	− 6.8–13.5	− 14.8–9.4	0.0	0.0
	Hernia dissection	4.0 (2.8)	1.5 (2.1)	− 5.1–12.6	− 5.1–7.5	3.7	0.0
	Suturing	4.7 (2.8)	1.9 (2.7)	− 17.8—17.9	− 10.4–10.8	0.5	0.7
	Preparing the mesh	1.1 (1.6)	2.0 (2.7)	− 2.9–5.1	− 3.8–6.0	0.0	0.0
	Mesh deployment	3.2 (2.9)	−0.8 (2.3)	− 4.1–11.8	− 9.9–6.2	1.1	0.1
	Assisting	3.5 (2.1)	−0.1 (2.6)	− 8.7–12.5	− 9.3–9.8	0.0	0.0
	Total	2.8 (2.8)	−1.4 (2.3)	− 17.5—17.8	− 15.8–8.3	21.9	0.9
Nondominant shoulder abduction	Access	13.5 (9.2)	5.8 (6.9)	− 18.1—57.1	− 13.9–46.3	3.3	1.3
	Hernia dissection	12.6 (8.0)	6.8 (7.1)	− 10.8—56.5	− 8.7–44.9	3.6	1.0
	Suturing	15.8 (9.2)	13.3 (8.4)	− 15.0—63.5	− 9.7–53.9	4.4	3.1
	Preparing the mesh	18.5 (6.5)	3.1 (6.3)	4.0–42.8	− 7.6–40.3	5.4	0.2
	Mesh deployment	13.9 (9.7)	7.3 (10.2)	− 4.9–54.7	− 14.7–51.1	14.7	2.2
	Assisting	12.6 (9.5)	8.0 (7.5)	− 36.0—69.0	− 12.4–52.8	1.1	0.4
	Total	12.3 (9.1)	12.4 (7.0)	− 30.7—77.1	− 10.9–62.2	50.9	23.2
Nondominant shoulder flexion	Access	31.6 (15.5)	22.9 (6.6)	− 15.5—114.1	3.1–64.4	38.0	3.5
	Hernia dissection	26.2 (14.0)	27.5 (8.0)	− 17.4—79.1	5.6–54.7	22.5	13.6
	Suturing	23.5 (12.9)	23.2 (10.1)	− 35.9—98.9	− 2.9–70.0	24.1	12.4
	Preparing the mesh	13.5 (8.9)	17.0 (6.1)	− 6.0–50.7	0.0–37.8	1.1	0.0
	Mesh deployment	24.1 (12.7)	26.0 (9.6)	− 7.9–68.2	3.8–62.7	51.4	15.9
	Assisting	27.0 (15.3)	22.5 (9.0)	− 53.3—125.2	− 9.9–68.1	11.1	4.8
	Total	21.6 (13.7)	18.5 (8.0)	− 63.9—105.0	− 18.6–73.8	70.0	48.6
Dominant shoulder abduction	Access	14.4 (10.4)	10.0 (7.7)	− 10.6—65.6	− 7.8–51.4	30.0	3.4
	Hernia dissection	18.0 (9.7)	7.6 (9.0)	− 10.2—58.0	− 14.2–52.1	9.6	5.5
	Suturing	17.5 (9.1)	9.3 (7.3)	− 13.6—65.6	− 9.9–43.7	5.4	1.8
	Preparing the mesh	15.7 (7.3)	17.0 (10.3)	− 0.7–50.5	0.5–54.9	1.9	3.4
	Mesh deployment	15.0 (8.5)	9.4 (8.1)	− 7.1–54.6	− 7.5–45.2	7.4	1.1
	Assisting	15.3 (13.4)	8.0 (8.5)	− 52.5—69.7	− 16.7–55.5	3.0	0.9
	Total	14.6 (10.1)	8.4 (7.1)	− 33.5—76.1	− 18.5–54.2	55.7	24.2
Dominant shoulder flexion	Access	29.6 (18.0)	17.2 (7.6)	− 15.1—119.3	− 4.1–67.3	41.8	4.4
	Hernia dissection	21.4 (13.7)	19.4 (9.5)	− 13.0—75.7	− 32.2–71.7	21.9	10.9
	Suturing	19.7 (11.6)	13.4 (9.0)	− 41.4—73.3	− 11.8–51.8	18.6	13.3
	Preparing the mesh	15.7 (10.0)	19.9 (15.4)	− 8.7–62.7	0.0v74.5	2.6	3.2
	Mesh deployment	23.3 (13.6)	20.1 (8.1)	− 15.9—70.8	1.7v58.6	53.3	5.5
	Assisting	20.5 (17.7)	14.7 (8.6)	− 60.9—134.3	− 8.3–76.1	6.9	1.0
	Total	20.4 (15.0)	22.5 (8.6)	− 62.7—125.2	− 14.6–90.8	76.8	37.7

Laparoscopic hernia repair							
Posture Angle	Segment	Control Mean (SD)	Exoskeleton Mean (SD)	Control Range	Exoskeleton Range	Control Percent	Exoskeleton Percent
Neck flexion	Trocar insertion	18.0 (5.4)	14.6 (4.4)	5.4–28.5	4.8–24.6	100.0	100.0
	Access	13.0 (2.8)	13.3 (3.5)	6.8–24.0	6.6–24.6	87.0	79.3
	Hernia dissection	12.6 (3.4)	11.7 (2.7)	4.5–25.3	2.2–24.1	97.2	62.9
	Suturing	16.6 (5.4)	19.2 (4.7)	1.0–27.5	6.0–28.0	84.8	98.5
	Preparing the mesh	19.3 (4.3)	16.6 (3.5)	7.4–25.1	8.1–22.6	90.3	74.2
	Mesh deployment	14.3 (3.6)	12.5 (3.9)	6.9–25.8	− 2.0–24.3	95.0	57.2
	Assisting	13.0 (3.3)	9.1 (2.5)	2.8–25.7	3.0–21.7	100.0	64.4
	Total	14.0 (5.4)	12.6 (4.1)	− 3.1–30.1	− 2.1–26.0	100.0	100.0
Trunk flexion (L5/S1)	Trocar insertion	−1.6 (2.0)	−2.3 (1.2)	− 6.5–4.4	− 6.1–2.2	0.0	0.0
	Access	0.6 (1.3)	−1.0 (1.1)	− 3.1–4.8	− 4.2–2.5	0.0	0.0
	Hernia dissection	−0.2 (1.6)	−0.2 (1.3)	− 5.3–6.8	− 8.8–9.6	0.0	0.9
	Suturing	−0.6 (1.8)	0.6 (2.0)	− 8.6–7.5	− 6.0–6.3	0.1	0.0
	Preparing the mesh	−0.6 (2.1)	−1.2 (1.6)	− 4.4–4.6	− 4.5–3.1	0.0	0.0
	Mesh deployment	−0.1 (1.6)	−1.2 (2.5)	− 4.2–5.3	− 16.6–15.2	0.0	0.1
	Assisting	−1.8 (1.4)	−2.3 (1.2)	− 5.9–5.4	− 15.4–10.0	0.0	0.0
	Total	−0.2 (2.0)	−1.5 (1.6)	− 11.2–11.5	− 14.9–14.0	0.2	1.5
Nondominant shoulder abduction	Trocar insertion	13.6 (7.6)	16.2 (6.8)	− 3.7–58.1	− 1.2–50.5	7.7	2.5
	Access	15.4 (7.7)	14.3 (6.0)	− 0.4–51.9	0.1–47.1	8.5	1.9
	Hernia dissection	16.2 (8.0)	11.1 (9.1)	− 4.0–58.7	− 19.6–41.6	9.5	18.6
	Suturing	13.7 (6.6)	15.5 (7.5)	− 7.4–51.9	− 8.6–57.3	3.3	3.6
	Preparing the mesh	14.1 (8.1)	11.6 (6.4)	− 1.4–44.3	− 1.5–37.5	0.5	0.3
	Mesh deployment	14.8 (8.3)	14.4 (12.0)	0.6–60.1	− 16.9–57.4	3.2	9.0
	Assisting	14.8 (7.3)	16.9 (8.7)	− 10.9–66.1	− 17.4–79.0	12.9	4.3
	Total	13.7 (9.0)	16.2 (8.9)	− 22.3–71.6	− 25.5–74.3	45.3	41.7
Nondominant shoulder flexion	Trocar insertion	16.6 (12.5)	12.0 (8.3)	− 16.1–74.6	− 5.9–51.2	16.7	3.4
	Access	12.8 (9.0)	12.2 (7.7)	− 9.9–51.6	− 4.9–63.7	13.3	2.0
	Hernia dissection	11.3 (8.0)	5.0 (9.7)	− 12.1–67.8	− 37.9–74.1	12.7	20.0
	Suturing	10.9 (12.0)	16.0 (9.8)	− 24.0–79.4	− 40.2–78.8	10.7	7.7
	Preparing the mesh	16.2 (15.0)	15.9 (10.8)	− 12.8–57.9	− 3.0–52.9	3.4	2.0
	Mesh deployment	16.3 (10.7)	10.7 (9.6)	− 9.9–69.4	− 22.2–45.6	13.5	6.2
	Assisting	10.4 (15.8)	15.0 (11.7)	− 24.7–89.3	− 97.4–154.8	29.6	3.5
	Total	14.7 (13.1)	11.9 (10.7)	− 39.2–92.2	− 68.6–117.1	66.1	43.9
Dominant shoulder abduction	Trocar insertion	20.0 (11.9)	19.3 (8.3)	− 7.0–67.5	2.6–60.3	26.3	9.8
	Access	19.7 (6.8)	25.5 (8.6)	3.2–50.9	7.6–59.7	16.8	18.6
	Hernia dissection	19.8 (7.6)	26.5 (9.9)	− 1.5–57.4	− 5.6–67.4	16.2	25.4
	Suturing	18.4 (9.8)	22.1 (7.9)	− 7.6–62.6	3.3–64.5	20.8	10.5
	Preparing the mesh	22.5 (10.7)	16.7 (6.9)	4.0–64.4	3.6–45.2	11.3	0.6
	Mesh deployment	20.7 (8.3)	19.8 (10.9)	2.9–63.8	− 12.9–61.3	9.8	19.8
	Assisting	19.8 (9.4)	25.0 (10.8)	− 24.4–69.8	− 26.8–65.0	23.1	16.4
	Total	19.9 (10.4)	22.5 (9.9)	− 30.4–77.4	− 23.6–71.5	74.4	58.7
Dominant shoulder flexion	Trocar insertion	16.2 (16.6)	15.0 (9.3)	− 20.4–96.0	− 10.3–61.3	35.2	8.2
	Access	18.4 (8.2)	15.7 (9.7)	− 5.2–62.6	− 12.0–58.4	16.3	17.9
	Hernia dissection	14.8 (9.2)	4.2 (13.4)	− 16.2–75.5	− 51.0–83.1	13.6	20.7
	Suturing	15.0 (12.7)	16.4 (9.8)	− 28.4–75.5	− 52.8–93.2	16.0	9.9
	Preparing the mesh	16.6 (14.3)	12.9 (8.9)	− 8.3–76.9	− 1.3–46.8	7.2	0.6
	Mesh deployment	18.4 (11.6)	16.3 (15.4)	− 23.8–72.1	− 48.0–82.1	12.8	15.6
	Assisting	12.2 (15.6)	11.9 (16.9)	− 63.4–108.2	− 83.1–70.0	26.8	19.8
	Total	17.4 (14.2)	14.0 (13.8)	− 70.6–119.1	− 68.4–88.8	77.9	55.1

#### Effects of exoskeleton

For Access, compared to control, there was a significant difference between mean neck flexion (control–exo mean difference [CEMD] = −7.76, *t* = −5.16, *p* < 0.01), trunk flexion (CEMD = 3.20, *t* = 2.93 *p* < 0.01), non-dominant shoulder abduction (CEMD = 7.66, *t* = 4.42, *p* < 0.01) and flexion (CEMD = 8.67, *t* = 2.64, *p* = 0.02), and dominant shoulder flexion (CEMD = 12.38, *t* = 3.28, *p* < 0.01). For Hernia dissection, there was a significant difference in neck flexion (CEMD = 12.38, *t* = -3.09, *p* = 0.01), non-dominant shoulder abduction (CEMD = 5.76, *t* = 2.59, *p* = 0.02), and dominant shoulder abduction (CEMD = 10.38, *t* = 4.01, *p* < 0.01). For Mesh deployment, there was a significant difference in neck flexion (CEMD = −7.63, *t* = −2.82, *p* = 0.02). For Suturing, there was a significant difference in neck flexion (CEMD = −6.86, *t* = −5.35, *p* < 0.01) and dominant shoulder abduction (CEMD = 8.13, *t* = 2.58, *p* = 0.01). For Assisting, there was a significant difference in neck flexion (CEMD = −8.35, *t* = −7.91, *p* < 0.01), trunk flexion (CEMD = 3.58, *t* = 4.30, *p* < 0.01), abduction in the non-dominant (CEMD = 4.59, *t* = 2.99, *p* < 0.01) and dominant shoulder (CEMD = 7.33, *t* = 5.08, *p* < 0.01), and flexion in the non-dominant (CEMD = 4.54, *t* = 2,53, *p* = 0.01) and dominant shoulder (CEMD = 5.81, *t* = 3.10, *p* < 0.01).

#### Laparoscopic hernia repair cases

Mean posture angles for laparoscopic cases can be found in (Fig. 3B) and descriptives of can be found in Table 5. The largest non-dominant shoulder flexion was during Trocar insertion and dominant shoulder flexion was during Access. The longest mean percentage of time spent in extreme non-dominant and dominant shoulder flexion was observed during Assisting and Trocar insertion. The largest neck and trunk flexion was during Preparing the mesh and Assisting. The longest mean percentage of time spent in extreme neck and trunk flexion was during Trocar insertion and Suturing respectively.

#### Effects of exoskeleton

For Trocar insertion, there was a significant difference in neck flexion (CEMD = 3.42, *t* = 2.43, *p* = 0.03). For Hernia dissection, there was a significant difference in the dominant shoulder (CEMD = 10.52, *t* = 2.28, *p* = 0.04). For Preparing mesh, there was a significant difference in the dominant shoulder (CEMD = 5.80, *t* = 2.25, *p* = 0.04). For Assisting, there was a significant difference in neck flexion (CEMD = 3.88, *t* = 2.30, *p* = 0.04) and non-dominant shoulder flexion (CEMD = −4.61, *t* = −2.52, *p* = 0.03).

### Self-reported metrics

Results for the self-reported discomfort show mean discomfort being rated between 0 and 1 for all evaluated muscles for both control exoskeleton conditions across open and laparoscopic cases. The usability of the shoulder-support exoskeleton was rated a 74.4 / 100 (8.7) for open hernia repair cases and a 78.1 / 100 (8.3) for laparoscopic hernia repair cases.

## Discussion

This study characterized the muscle activity experienced and posture adopted during key segments of hernia repair procedures. Similar to previous literature, this study found that open procedures led to higher muscle activity in the UT and ES compared to laparoscopic procedures [18, 19]. Considering open cases, Mesh deployment was likely the largest contributor since participants spent the most time in this segment and recorded the highest muscle activity. Laparoscopic cases had largely stable muscle activity with some spikes in the ES being recorded during Preparing mesh since this is when attendings stepped away from the OR table to cut out the mesh. Since this is also the shortest segment in the laparoscopic cases, future interventions may aim to support alternative steps such as Mesh deployment or Access.

Considering the effects of a shoulder-support exoskeleton, this study found that during open procedures the exoskeleton led to a reduction in UT muscle activity and shoulder and trunk flexion at the expense of increased ES muscle activity and neck flexion; laparoscopic cases largely saw an increase in UT and ES muscle activity and a decrease in neck, shoulder, and trunk flexion. This study echoes and challenges previous work where it was found that a shoulder-support exoskeleton also reduced 50th %ile left trapezius muscle activation; yet, contrary to this study, the exoskeleton used by Cha et al. [3] also led to a reduction in right ES muscle activity [3]. Some studies have also found that when exoskeletons were used by surgeons during procedures it led to decreased neck, shoulder, and arm discomfort [8]. Although the device in this study was rated as having “good” usability, surgeons reported no difference in self-reported discomfort when using the exoskeleton which coincides with other literature [15].

This study did include a few limitations. The first of which is that the attendings included in this study were all from the same healthcare institution, which may limit the generalizability of their results across a larger population sample. Another limitation was that the case segments were denoted verbally by the attending surgeon and through observation of the researcher. Due to this, there may have been instances where the attending surgeon or researcher noted the change in segment late which may have affected the exact start and end of each segment. However, a researcher would often verbally check-in with the surgeon to confirm any changes in case segments. Additionally, attendings completed a range of inguinal and ventral cases which each have their own physical demands. As such, these case variations may have influenced their recorded muscle activity and posture throughout the case. Further, as this was a training institution, attending surgeons and surgical fellows would switch roles as leads during the case and as such the recorded physical exposure may not be representative of a single attending completing the entire case. The side the attending was on, table height, the location of the hernia, and their handedness may also have affected muscle activity and posture results as this may have created instances where the attending was under greater demands due to operating on their non-dominant side or dissecting a hernia that was difficult to reach. Finally, the exoskeleton may have been another limitation as it was designed primarily for manufacturing applications and thus may not be ideal to support the postures adopted during surgical procedures which are often influenced by sterility constraints and operating room ergonomics.

## Conclusion

This study stratified the unique muscle activity and posture adopted during key case segments of open and laparoscopic hernia repair procedures. Additionally, it found that a shoulder-support exoskeleton may be more suited for the physical demands of open hernia repairs and detrimental during laparoscopic cases. As such, exoskeletons still offer a promising future to support surgeons and reduce the risk of developing WMSDs.
